# Snowbird Thanksgiving Miracle

**DOI:** 10.1097/CNJ.0000000000001305

**Published:** 2026-03-02

**Authors:** Jake Loutensock, Adrianna Watson

**Affiliations:** **Jake Loutensock** is a senior nursing student who aspires to become a certified registered nurse anesthetist (CRNA) to help and change people's lives for the better. During the COVID-19 pandemic, he helped manage and operate testing and vaccine clinics in his home state.; **Adrianna Watson, PhD, RN, CCRN, TCRN,** is an assistant professor and nurse researcher at Brigham Young University where she mentors undergraduate nursing students in research and clinical practice.

On November 29, 2024, the day after Thanksgiving, I decided to go skiing with my family. We got to the slopes at about 7:30 a.m. Although we hadn't planned to, we were guided to take a tram to the very top of the mountain, accessing steep and challenging terrain. As we descended on the beautiful morning snow, I saw a man in front of me take a face-first fall on a run just under the chairlift.

As I approached, two bystanders were standing nearby. One bystander said, “He isn't responding. I think he got knocked out.” These words triggered my nursing response. I ripped off my skis, threw off my gloves, and yelled for one person to call 911 and the second person to get a hold of ski patrol and an emergency provider. Then I began to assess the skier.

He was face down on the snow in a patch of frozen blood. “SIR! Can you hear me? Are you awake? SIR! I need you to respond to me,” I shouted. Nothing. I rolled him over onto his back; his face was covered in blood, and he had no respirations. I dug my fingers into his neck to find a carotid pulse. Nothing.

I began chest compressions as a man came alongside me, saying he was a physician. He took over cardiopulmonary resuscitation (CPR) for me when I got fatigued. At the 2-minute mark, we reassessed and found a steady carotid pulse. At this point the Snowbird Ski Patrol was on scene, so the physician and I transitioned the care into their hands. I walked over to the side of the run with a ski patroller and submitted a witness statement. The ski patrol loaded the man into a toboggan and transported him to the nearest AirMed landing spot on the mountain.

Soon I heard the rotor blades of the incoming helicopter. In the chaos of the event, my gloves seemed to have been taken into the toboggan with the patient. A ski patroller offered me his gloves as we skied down to the base area. He offered me some hot chocolate while we sat and talked for a minute.

A few hours later, I received a call from someone at the ski slope. The caller thanked me for my response and said that the quick initiation of CPR had saved the skier's life. She reported that it appeared as if the skier was going to make it. I breathed a huge sigh of relief.

I am deeply grateful that I was at that place with the right set of skills. As I reflect, I realize it wasn't just about skill or training: It was about choosing to act. The parable of the Good Samaritan (Luke 10:25-37) teaches that love isn't passive; it requires action. The priest and the Levite had their reasons for passing by, but the Samaritan stopped. He saw the suffering of a stranger and took responsibility for the man's care. That morning, I could have assumed someone else would step in. I could have hesitated. But my faith and values told me otherwise. Loving my neighbor meant stopping, assessing, and doing everything I could to give him a fighting chance.

I think of the woman in Luke 8:43-48 with the issue of blood who reached out to Jesus in faith. Jesus stopped. He noticed her, spoke to her, and acknowledged her need. He wasn't just focused on the crowd or his destination. He saw the one in need. That story reminds me that, in moments of crisis, we are called not just to see suffering, but to respond with compassion and action.

Looking back, I see how my education prepared me with clinical knowledge as well as the mindset, resilience, and purpose to step forward in a moment of high stress, uncertainty, and chaos. More than anything, I am thankful that the man is recovering, and I am humbled that I was able to be part of something greater than myself...a moment where faith, preparation, and compassion came together in a way I will never forget.

